# Enhancing Supercapacitor Electrochemical Performance with 3D Printed Cellular PEEK/MWCNT Electrodes Coated with PEDOT: PSS

**DOI:** 10.1021/acsomega.4c04576

**Published:** 2024-07-23

**Authors:** Athul C. S. Chandran, Johannes Schneider, Reshma Nair, Buchanan Bill, Nikolaj Gadegaard, Richard Hogg, Shanmugam Kumar, Libu Manjakkal

**Affiliations:** † School of Computing and Engineering & the Built Environment, Edinburgh Napier University, Merchiston Campus, Edinburgh EH10 5DT, U.K.; ‡ James Watt School of Engineering, University of Glasgow, Glasgow G12 8QQ, U.K.; § School of Engineering and Applied Science, Aston University, B4 7ET Birmingham, U.K.

## Abstract

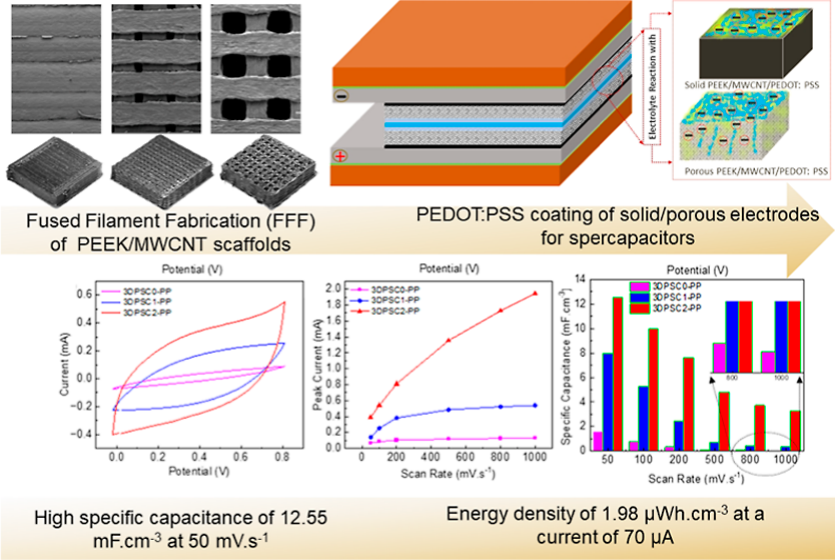

In this study, we examine the electrochemical performance of supercapacitor (SC) electrodes made from 3D-printed nanocomposites. These composites consist of multiwalled carbon nanotubes (MWCNTs) and polyether ether ketone (PEEK), coated with poly(3,4-ethylenedioxythiophene) polystyrene sulfonate (PEDOT:PSS). The electrochemical performance of a 3D-printed PEEK/MWCNT solid electrode with a surface area density of 1.2 mm^–1^ is compared to two distinct periodically porous PEEK/MWCNT electrodes with surface area densities of 7.3 and 7.1 mm^–1^. To enhance SC performance, the 3D-printed electrodes are coated with a conductive polymer, PEDOT:PSS. The architected cellular electrodes exhibit significantly improved capacitive properties, with the cellular electrode (7.1 mm^–1^) displaying a capacitance nearly four times greater than that of the solid 3D-printed electrode-based SCs. Moreover, the PEDOT:PSS-coated cellular electrode (7.1 mm^–1^) demonstrates a high specific capacitance of 12.55 mF·cm^–3^ at 50 mV·s^–1^, contrasting to SCs based on 3D-printed cellular electrodes (4.09 mF·cm^–3^ at 50 mV·s^–1^) without the coating. The conductive PEDOT:PSS coating proves effective in reducing surface resistance, resulting in a decreased voltage drop during the SCs’ charging and discharging processes. Ultimately, the 3D-printed cellular nanocomposite electrode with the conductive polymer coating achieves an energy density of 1.98 μW h·cm^–3^ at a current of 70 μA. This study underscores how the combined effect of the surface area density of porous electrodes enabled by 3D printing, along with the conductivity imparted by the polymer coating, synergistically improves the energy storage performance.

## 1 Introduction

The demand for rechargeable electrochemical energy storage devices, such as batteries and supercapacitors (SCs), has surged across various applications for portable electronic gadgets.^1−4^ The combination of a rapid increase in the world’s population and technological advancements has substantially heightened the demand for energy storage devices.^5^ Electrochemical storage facilities stand out among various types, drawing attention for their maximal reliability, specific cost-effectiveness, and adaptable capacities.^6,7^ While rechargeable batteries are more widely utilized due to their high energy storage capability and repeatability, they come with drawbacks such as lower power densities, a reduction in cycle life expectancy, extended charging periods, thermal management issues, and environmental safety concerns.^8−11^ In overcoming these challenges and catering to applications requiring low-powered sensing, rapid power delivery, and prolonged lifetimes, SCs hold significant importance due to their distinctive properties.^5,12,13^ The key requirements for SC electrode materials are high conductivity or low impedance, a substantial electrochemically active surface area, and porous structures demonstrating excellent cyclic stability.^14^ The development of SCs involves various fabrication methods, materials, and architectures, offering diverse avenues for advancement.^15−19^

Various printing technologies, including screen printing, 3D printing, and inkjet printing, are widely employed for the cost-effective production of electrodes in energy storage devices.^20−23^ However, the challenge lies in achieving efficient material architectures that reduce material waste and use low-cost materials for electrode fabrication.^24^ Additive manufacturing, specifically 3D printing, is extensively acknowledged for addressing these challenges in fabricating energy storage electrodes with diverse architecture.^25−27^ The anticipated benefits of 3D-printed energy storage devices include high energy and power density, lightweight design, rapid charging rates, and extended lifetime.^28,29^ A variety of materials, such as polypyrrole@Ag,^30^ MXene Sediment ink,^24^ WO_3_ anodes and Prussian blue cathodes,^31^ and Ti_3_C_2_T_*x*_ MXene/Cellulose Nanofiber,^32^ have been utilized in the 3D printing of SCs, showcasing remarkable performances due to their high conductivity, specific capacity, and pseudo capacitance behavior. As compared to other thin film methods of fabrication, the major issue of the 3D printed electrodes is the additives in the material which reduce the conductivity and hence performance.^33^ The limited availability of material options for 3D printing poses another challenge in developing high-energy storage materials compared to screen printing or solution coating methods.

Introducing pores into electrodes offers several advantages, including enhancing the mass loading of high-performance active materials, facilitating effective ion diffusion and electron transport, and expediting the reaction kinetics.^25^ Additionally, porous electrodes also impart strain tolerance. Therefore, this study explores the electrochemical performances of lattice electrodes comprising polyether ether ketone (PEEK) and multi-walled carbon nanotubes (MWCNTs), processed through fused filament fabrication (FFF) for SC applications. This study introduces PEEK/MWCNT electrode materials with a 3D-printed lattice (periodically porous) structure, distinct from traditional fully dense (solid) PEEK nanocomposites. The lattice design increases surface area, reduces weight, enhances strain tolerance, improves cyclability, and boosts the energy/power density. Such architected electrodes cannot be produced with conventional manufacturing methods, highlighting the innovative use of 3D printing. The porous electrode design enhances the electrode surface area density and provides more electrolyte-accessible areas for electrochemical reactions. By comparing the electrochemical performance, solid 3D-printed PEEK/MWCNT electrodes with a surface area density of 1.2 mm–^1^ demonstrate superior energy storage capabilities compared to cellular electrodes with surface area densities of 7.3 and 7.1 mm–^1^. To improve the conductivity of the electrode surface for ion interaction and mass loading into the material pores, the PEEK/MWCNT-based 3D printed electrodes (both solid and porous) were coated with poly(3,4-ethylenedioxythiophene) polystyrenesulfonate (PEDOT:PSS) (PP). This conductive polymer coating partially covered both the bulk electrode surface and the inner walls of the pores. Based on the prepared 3D-printed electrodes and the modified electrodes, two sets of symmetric SCs (3DPSC and 3DPSC-PP) are developed, and their performances are compared. A detailed electrochemical performance analysis, including cyclic voltammetry (CV), electrochemical impedance spectroscopy (EIS), and galvanostatic charging discharging (GCD), revealed the excellent performance of porous and conductive polymer-coated porous electrodes. The synergistic effect of increased surface area density of lattice materials and the conductivity of the polymer coating resulted in an enhanced energy storage performance.

## 2 Experimental Methods

### 2.1 Design and Additive Manufacturing of Fully Dense and Porous Electrodes

The electrodes’ computer-aided design (CAD) model was generated using SolidWorks (Dassault Systèmes) to create architected cellular structures based on periodically arranged unit cells, serving as representative elements. The design aimed to incorporate a periodic porous microstructure with a high surface area density (surface area to volume ratio in mm^2^/mm^3^) within a confined design space measuring 10 × 10 × 2.4 mm^3^. Two cellular designs were considered, featuring different pore sizes, resulting in relative densities, **ρ̅**, of 73 and 55%, representing the ratio of the cellular material’s density to the parent material’s density. This equated to surface area densities of 7.3 and 7.1 mm^–1^, respectively, compared to the 1.2 mm^–1^ of the solid electrode.

The fabrication utilized FFF for both the lattices and the bulk material. FFF involves the layer-by-layer extrusion of filament onto a build plate through a heated nozzle. The printhead, comprising the heated nozzle, can move in-plane, while the build plate moves out of plane to make room for subsequent layers. The cellular design was arranged to be manufactured with a single, continuous nozzle movement, ensuring precise and consistent printing results within the confined build space and achieving the highest resolution possible for the chosen printing technique and setup. The walls of the cellular structures had a thickness of approximately 480 μm, corresponding to the standard extrusion width. The minimum reliable extrusion width, and consequently the feature size achieved in the *x*–*y* plane with this configuration, is approximately 0.48 mm (using a nozzle diameter of 0.4 mm). The continuous nozzle movement significantly improves print quality, akin to the so-called vase mode. This method eliminates issues commonly associated with stop-and-go printing, such as oozing, stringing, and inconsistencies when the nozzle has to bridge gaps or extrude intricate features at these fine length scales. This approach ensures precise and consistent results, maximizing the resolution achievable with our chosen printing technique and setup.

**Figure 1:**
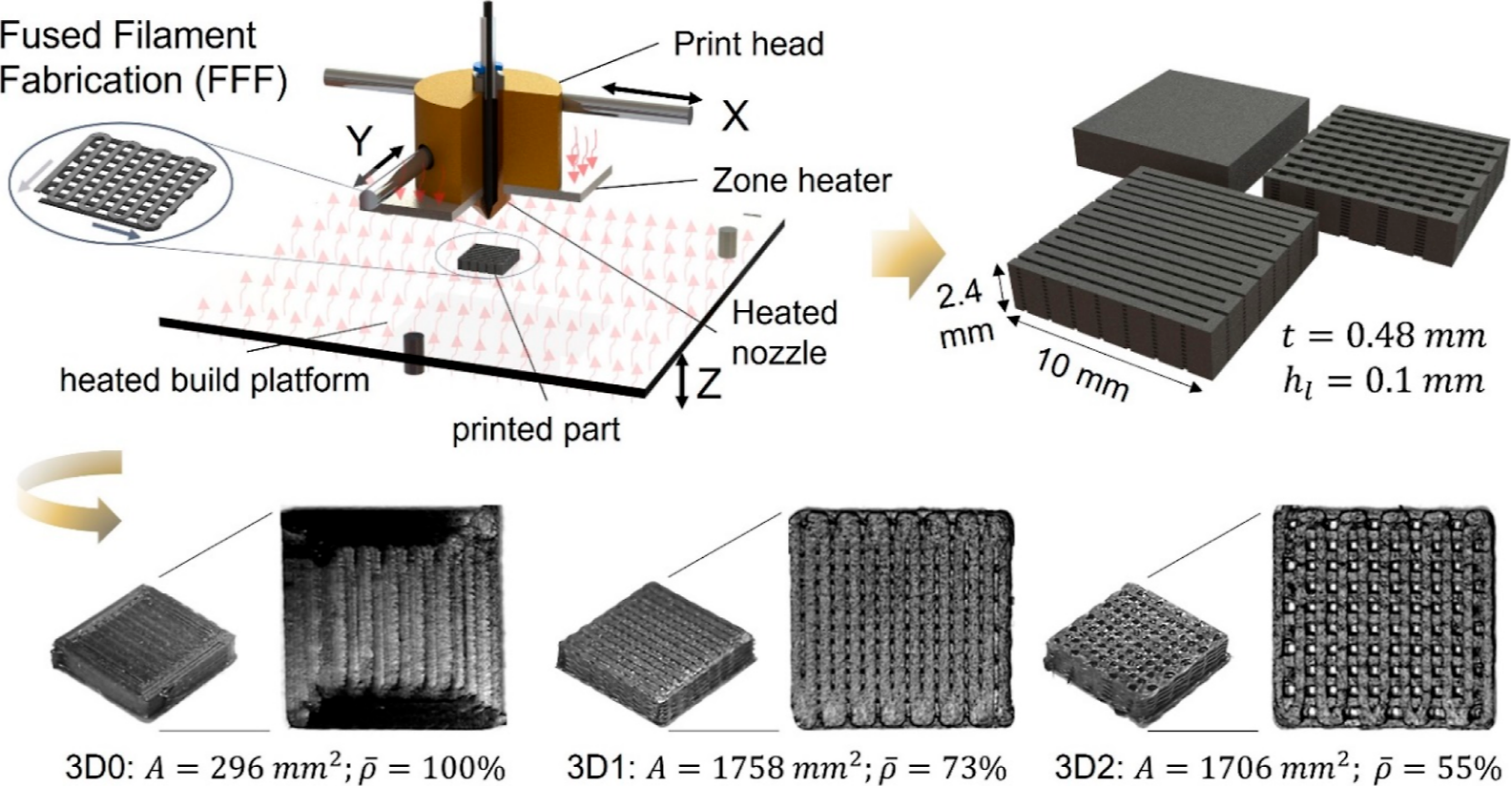
Schematic representation of the FFF process, illustrating the realization of solid and cellular electrodes for SCs, showcasing surface area, *A*, relative density, **ρ̅** extrusion width, *t*, and layer height, *h*_l_.

Figure 1 illustrates the FFF process for fabricating bulk and cellular specimens, showcasing their parameters, and including a nozzle movement diagram for the first two layers of the cellular specimens. The bottom row illustrates the solid 3D printed PEEK/MWCNT electrode (3D0) with an area of 296 mm^2^, alongside the cellular porous electrodes (3D1 and 3D2) with areas of 1758 and 1706 mm^2^, respectively, demonstrating varying pore structures. The surface areas of the porous electrodes were determined in SolidWorks using the “Mass Properties” tool, which calculates the total surface area by summing the areas of all the small geometric faces that make up the complex geometries of the cellular architectures.

We employed commercially available 3DXSTAT ESD-Safe PEEK filament feedstock (3DXTECH), which consists of Victrex PEEK and MWCNTs. PEEK, a high-temperature thermoplastic polymer, showcases exceptional mechanical properties, including strength, stiffness, and toughness, surpassing standard plastics such as acrylonitrile butadiene styrene (ABS) or PLA. With a relatively high melting temperature of 343 °C, PEEK necessitates the use of a specialized high-temperature polymer 3D printer. For the fabrication of both bulk and cellular specimens in this study, we utilized an Apium P220 3D printer (Apium Additive Manufacturing GmbH). The printer was equipped with a heated build plate, and an additional zone heater was employed as a targeted heat source from the top to enhance the crystallinity of the printed parts, ensuring a high surface quality and improved layer adhesion. The printing temperature was set at 420 °C, while the bed and zone heater temperatures were maintained at 150 °C. The standard print speed was configured to 1600 mm/min, with a layer height of 100 μm. To eliminate any moisture content, the feedstock filament underwent drying at 60 °C for a minimum of 24 hours before the printing process.

### 2.2 Fabrication of SCs

**Figure 2:**
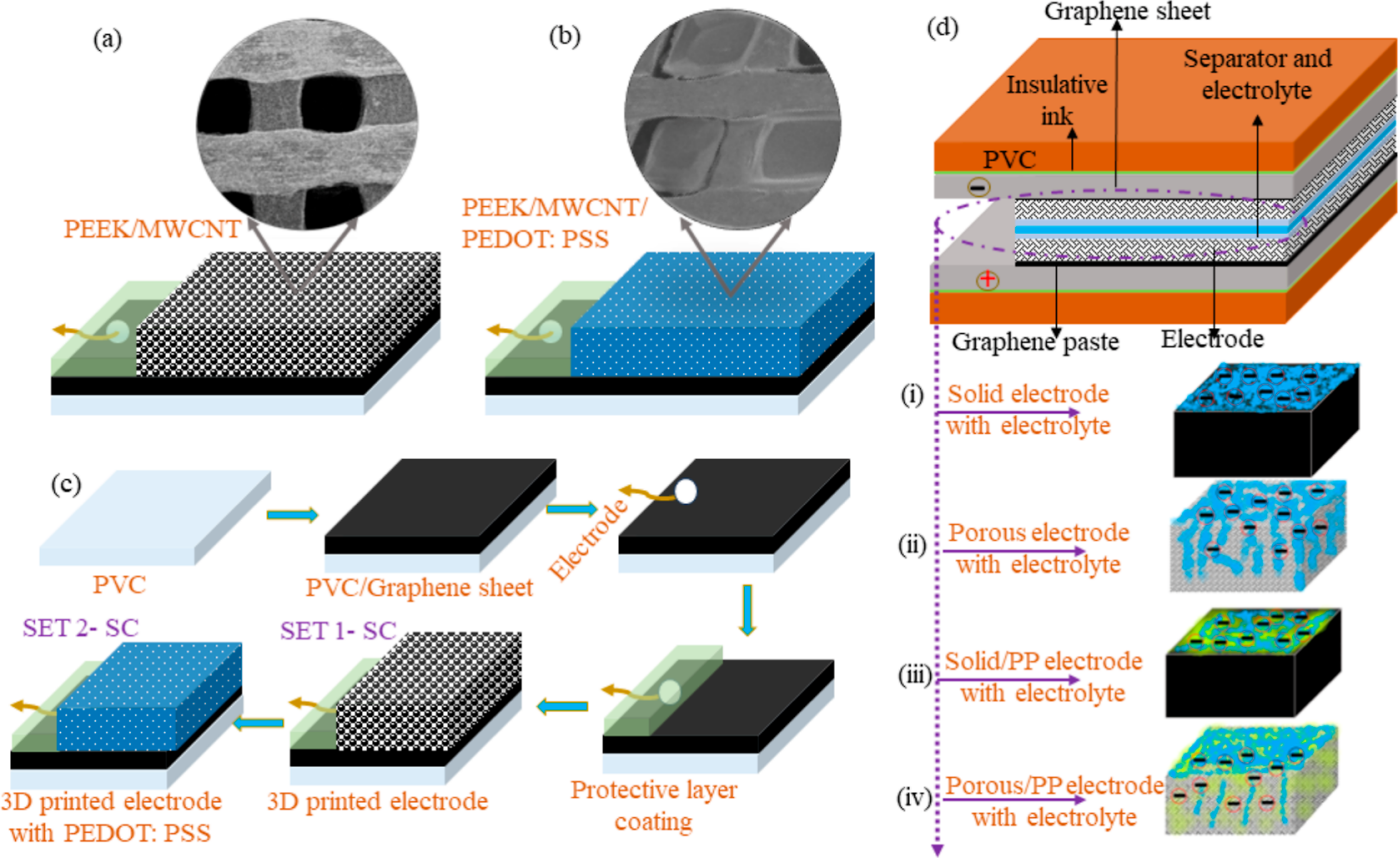
Images of schematic representation of fabricated electrodes for SCs with (a) 3D printed electrode-based PEEK/MWCNT (b) PEDOT: PSS coated 3D printed electrode (c) schematic representation of the fabrication steps of 3DPSCs electrodes (d) schematic representation of the design of developed 3D SCs. (i) 3D0 (solid) electrode reaction with electrolyte (ii) 3D1 (porous) electrode reaction with electrolyte (iii) 3D0 with PEDOT: PSS electrode reaction with electrolyte and (iv) 3D1 porous electrode with PEDOT:PSS layer electrode reaction with electrolyte.

Herein, we designed two sets of SCs utilizing 3D-printed electrodes, as depicted in Figure 2a,b. For Set 1, we fabricated SCs based on the PEEK/MWCNT, resulting in the development of three SCs named 3DPSC0, 3DPSC1, and 3DPSC2. Figure 2c illustrates the fabrication process of SC electrodes using the printed electrodes. Initially, a multilayer graphene sheet (Graphene Supermarket US) was affixed to a PVC substrate using insulating ink (JE solution, UK) and heat-treated at 80 °C for 1 hr. Following the attachment of the graphene sheet, the 3D-printed electrodes were affixed on one side, and a wire was attached to the other side using carbon paste (JE solution, UK). The electrodes and wires underwent curing at 80 °C for 1 hr. A protective layer of isolating ink was applied on the top of the wire side and heat-treated at 80 °C for 1 hr.

For set 2 devices, on top of the bonded 3D-printed electrodes, a conductive polymer was deposited and heated at 80 °C for 30 min in an oven. Before coating, the dropping ink was prepared by mixing 5 wt % dimethyl sulfoxide (DMSO, Sigma-Aldrich) with PEDOT:PSS (PP) (Oscilla) using magnetic stirring to enhance the conductivity of PEDOT:PSS.^12^ The developed set 2 SCs were named 3DPSC0-PP, 3DPSC1-PP, and 3DPSC2-PP. Figure 2d provides a schematic representation of the fabricated SCs. In electrochemical analysis, both PEDOT: PSS and the 3D-printed electrode participated in the reaction.

For SC fabrication, polyester/cellulose (Techni Cloth, TX 612) cloth served as a separator, and the device was tested in a 6 M KOH electrolyte. The developed SCs were encapsulated using a cling film and plastic packaging for testing electrochemical performances. Figure 2d(i–iv) depicts the 3D-printed SC electrodes and their electrolyte distribution. The porous structure allows the electrolyte and its ions to be distributed on the surface or inner side of the 3D-printed electrodes, depending on their types. For the 3D0 electrode, ions are concentrated on the surface [Figure 2d(i)], while for porous electrodes 3D1 and 3D2, the electrolyte diffuses inside the electrode through the pores, resulting in ion distribution on both the surface and inside the electrode [Figure 2d(ii)]. Figure 2d (iii,iv) depict the ionic distribution on 3D0 and 3D1 electrodes coated with PEDOT: PSS.

### 2.3 Characterization of Electrodes and SCs

Scanning electron microscopy (SEM, TESCAN VEGA 3) was used for evaluating the surface morphology of the 3D printed electrodes and the surface-modified electrodes. The characteristics of the functional group formation of the 3D printed electrodes were measured by using Fourier-transform infrared spectroscopy (FTIR, PerkinElmer Spectrometer). The electrochemical performances of the SCs of both sets such as CV, EIS and GCD were evaluated using an electrochemical workstation (Ivium Stat).

All electrochemical measurements were carried out in an electrode system for symmetric SCs. The CV analysis of the 3PDSCs and 3PDSC–PP was carried out at a scan rate of 50–1000 mV s^–1^ in a potential range of 0–0.8 V. The specific capacitance of the device was measured from CV by using reported equations.^12^ The CV analysis was used for investigating the diffusion reaction and redox reaction of the developed SCs. EIS measurements of the SCs were carried out from 10 mHz to 100 kHz at sinusoidal signals of 10 mV. EIS measurements enable us to understand the ion exchange from the KOH electrolyte, charge transfer, equivalent series resistance (ESR), and the supercapacitive behavior of 3DPSCs and the 3DPSC-PP devices. The GCD measurements of the fabricated 3DPSCs were tested by using different current densities with a potential window of 0–0.8 V.

## 3 Results and Discussion

### 3.1 Morphological Analysis of 3D Printed Electrodes

**Figure 3:**
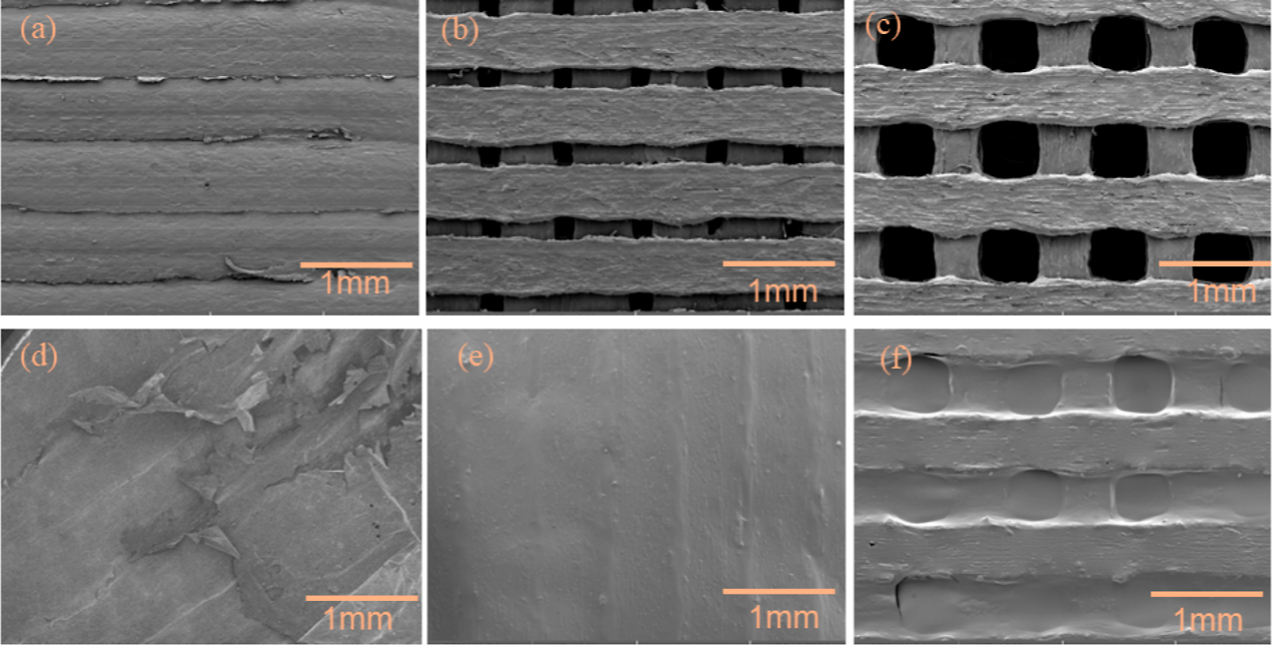
SEM images of 3D printed electrodes (a) solid electrode (3D0) (b) cellular porous electrodes with small pores and areas of 1758 mm^2^ (3D1) (c) cellular porous electrodes with larger pores and areas of 1706 mm^2^ (3D2). (d,e) PEDOT: PSS coated on the top of (d) 3D0 (e) 3D1 and (f) 3D2.

Figure 3a–c illustrates the surface morphologies of the 3D printed electrodes evaluated through SEM images, confirming the pore structure. The morphology illustrates an equal size and uniform shape of the pores for the 3D1 and 3D2 electrodes (Figure 3b,c). The SEM reveals a rough morphology for this printed solid electrode, as shown in Figure 3a. On the top of this 3D structure, PEDOT:PSS were coated to enhance the surface conductivity of the electrode. Figure 3d presents an SEM image of the 3D0 electrode after coating with PEDOT:PSS. The morphology indicates that the conductive polymer film is not strongly adhered to the solid structure 3D0. Figure 3e,f show the PEDOT:PSS coated on pores structure 3D1 and 3D2. It was observed that for porous samples the polymer film was coated as a membrane on a few pores, while others were without any coating or partially covered, as shown in Figure S1a,b in Supporting Information.

### 3.2 Electrochemical Performances of 3D-Printed Electrodes

**Figure 4:**
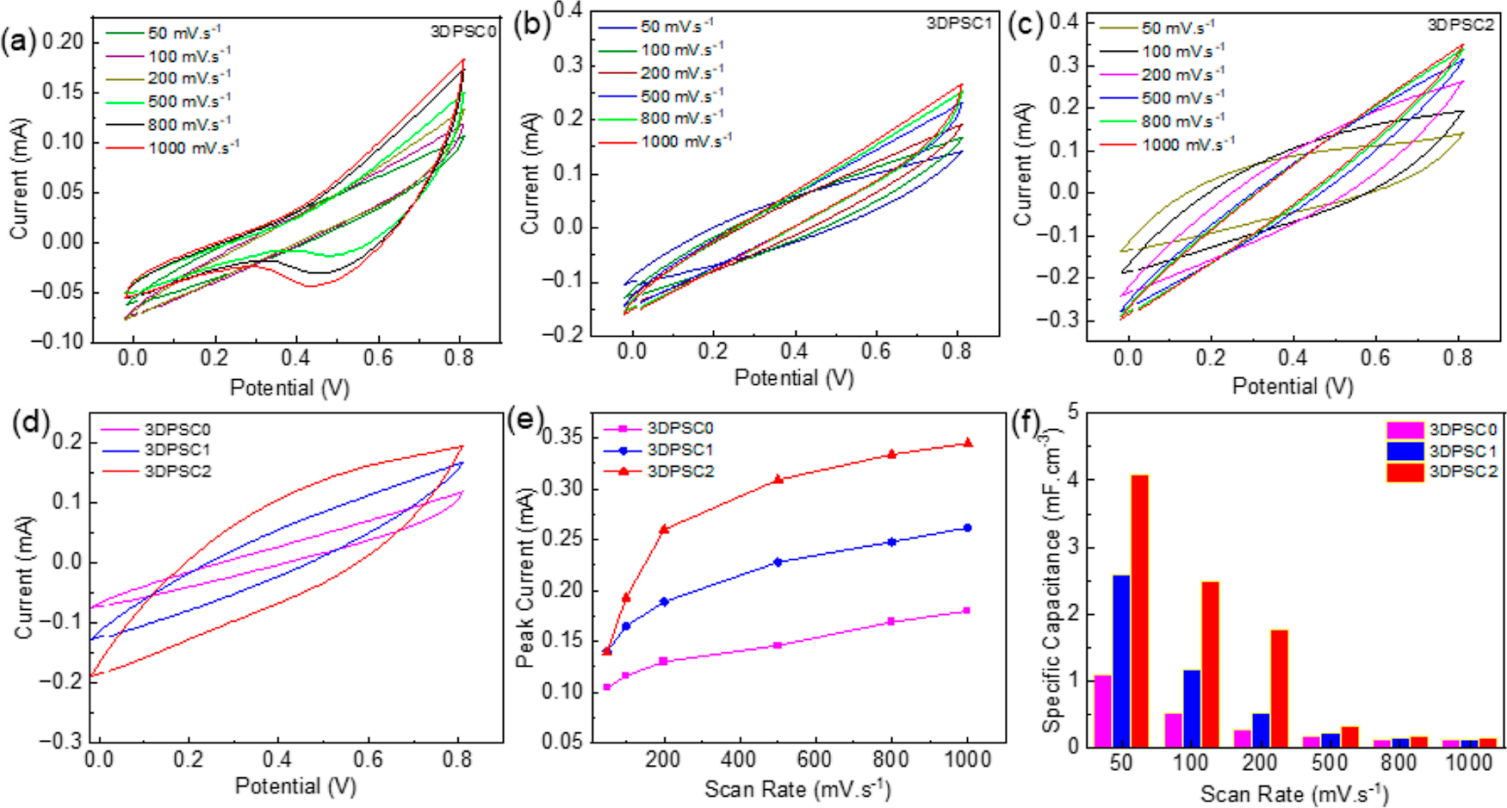
(a–c) CV curves with different scan rates for 3DPSC0, 3DPSC1, and 3DPSC2, respectively. (d) Comparison of CV curves observed for 3DPSCs at 100 mV·s^–1^. (e) Peak current with scan rate for various 3DPSCs. (f) Specific capacitance was measured from CV curves with different scan rates for various 3DPSCs.

Initially, we evaluated the electrochemical performance of the SCs fabricated using 3D0, 3D1, and 3D2 electrodes. The comparison of CV curves for different scan rates for 3DPSC0, 3DPSC1, and 3DPSC2 is presented in Figure 4a–c. A quasi rectangular shape in the CV data illustrates the pseudocapacitance influence of the electrodes during the reaction with electrolytes. The performances of SCs for a fixed scan rate of 100 mV·s^–1^ are compared and presented in Figure 4d, revealing a larger CV curve area for 3DPSC2. With increasing scan rate from 50 to 1000 mV·s^–1^, an increase in current appears in the CV, as shown in Figure 4e. The specific capacitance is measured from these CV data, and the value per unit volume is given in Figure 4f demonstrating that 3DPSC2 has high specific capacitance. At a scan rate of 50 mV s^–1^ the 3DPSC2 exhibited a specific capacitance of 4.09 mF·cm^–3^. The observed specific capacitance value for the porous sample (3D2) is almost twice as high as that for sample 3D1 and four times higher than that for the solid sample (3D0). The specific capacitance value illustrates that the structural porosity enhances the electrode surface area for electrolyte interaction. CV analysis for the devices was also carried out over different potential windows. The CV curves for 0.5, 0.8, and 1 V at 100 mV·s^–1^ for 3DPSC0, 3DPSC1, and 3DPSC2 are given in Figure S2 in the Supporting Information.

**Figure 5:**
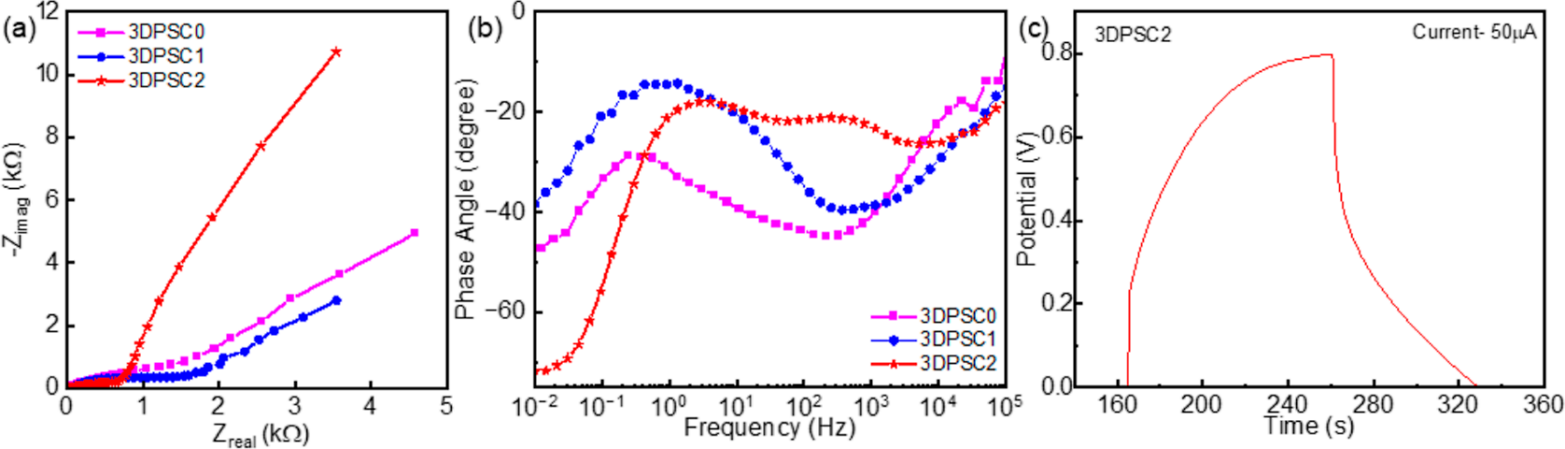
(a,b) Nyquist and Bode plot for 3DPSC0, 3DPSC1, and 3DPSC2. (c) GCD plot for 3DPSC2 at 50 μA.

The interaction of different porous architecture 3D printed electrodes with a KOH electrolyte was investigated using EIS analysis through the Nyquist and Bode plots shown in Figure 5a,b. The comparison of the Nyquist plot for the PEEK/MWCNT-based 3DPSCs is shown in Figure 5a. The Nyquist plot indicates that electrodes with electrolytes have diffusion-controlled Warburg capacitive behavior. A similar plot was observed for all SCs with variation in impedance values due to the change in the architecture of the electrodes. The ESR was observed from the high-frequency intercept of *Z*_real_ in the Nyquist plot. The observed variations in ESR values including 12 Ω (3DPSC0 at 120 kHz), 36 Ω (3DPSC1, 105 kHz), and 42 Ω (3DPSC2, 105 kHz) predict that 3DPSC2 have lower ESR value. The absence of a semicircle in the high-frequency range in Nyquist plots implies better pore accessibility for the ions in the electrolyte during the electrochemical reaction. The 3D printed electrodes are based on multilayer printing and are partially conductive, which is revealed from the multiple peaks in the Bode phase angle plot in Figure 5b. In an ideal capacitor at low frequency, the phase angle will be −90° and here at low frequency, the device illustrates capacitive behavior. The phase angle illustrates −47° for 3DPSC0, −38° for 3DPSC1 and −71° for 3DPSC2. The lower phase shift in the low-frequency range compared to −90° of an ideal capacitor is attributed to the pseudocapacitance of the electrode.^34^

Figure 5c shows the GCD analysis for 3DPSC2 at 50 μA current and similar for 3DPSC0 and 3DPSC1 illustrated in Figure S3a,b in Supporting Information. When compared with the triangular shape of the GCD curve of an electrochemical double layer SCs, the 3DPSCs exhibited a slight deviation, which could be due to the presence of pseudocapacitance of the electrode. From the GCD curve, we noticed an IR drop for each device and are 120 mV for 3DPSC0, 90 mV for 3DPSC1, and 211 mV for 3DPSC2. This high value of IR_drop_ could reduce energy storage and lead to power loss for the device. The Coulombic efficiency of the 3DPSCs was found to be 67, 76 and 70% , respectively, for 3DPSC0, 3DPSC1, and 3DPSC2. The performances of the devices including specific capacitance, energy density, and power densities were measured by considering the IR drop in the equations as given below.
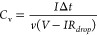
1
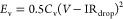
2
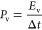
3

Where *C*_v_ is the specific capacitance in volume, *I* is the applied current for GCD analysis, Δ*t* is the discharge time, *V* is the voltage window, *v* is the volume of the electrode, IR_drop_ is potential drop, and *E*_v_ is the energy density and *P*_v_ is the power density. The measured specific capacitance of the 3DPSCs is illustrated in Figure S4a in Supporting Information. From the analysis, it was found that 3DPSC2 demonstrates a comparatively high specific capacitance of 23.11 mF·cm^–3^. The energy and power densities of 3DPSC2 are 1.11 μW·h·cm^–3^ and 59.47 μW·cm^–3^, respectively, and if observed the value of slightly less than compared to 3DPSC1 which is 1.27 μW·h·cm^–3^ (energy density) and 75.90 μW·cm^–3^ (power density). The variation of energy and power densities of the developed 3DPSCs is illustrated in Figure S4b,c in Supporting Information. Even though the 3DPSC2 illustrates high specific capacitance, the energy and power densities are slightly lower compared with 3DPSC1 which could be due to the larger IR_drop_. Due to the high thickness and high resistance, the device expected some dead volume of the material, which is not involved in the electrochemical reactions.

### 3.3 Electrochemical Performances of 3D-Printed Electrodes Coated with PEDOT:PSS

**Figure 6:**
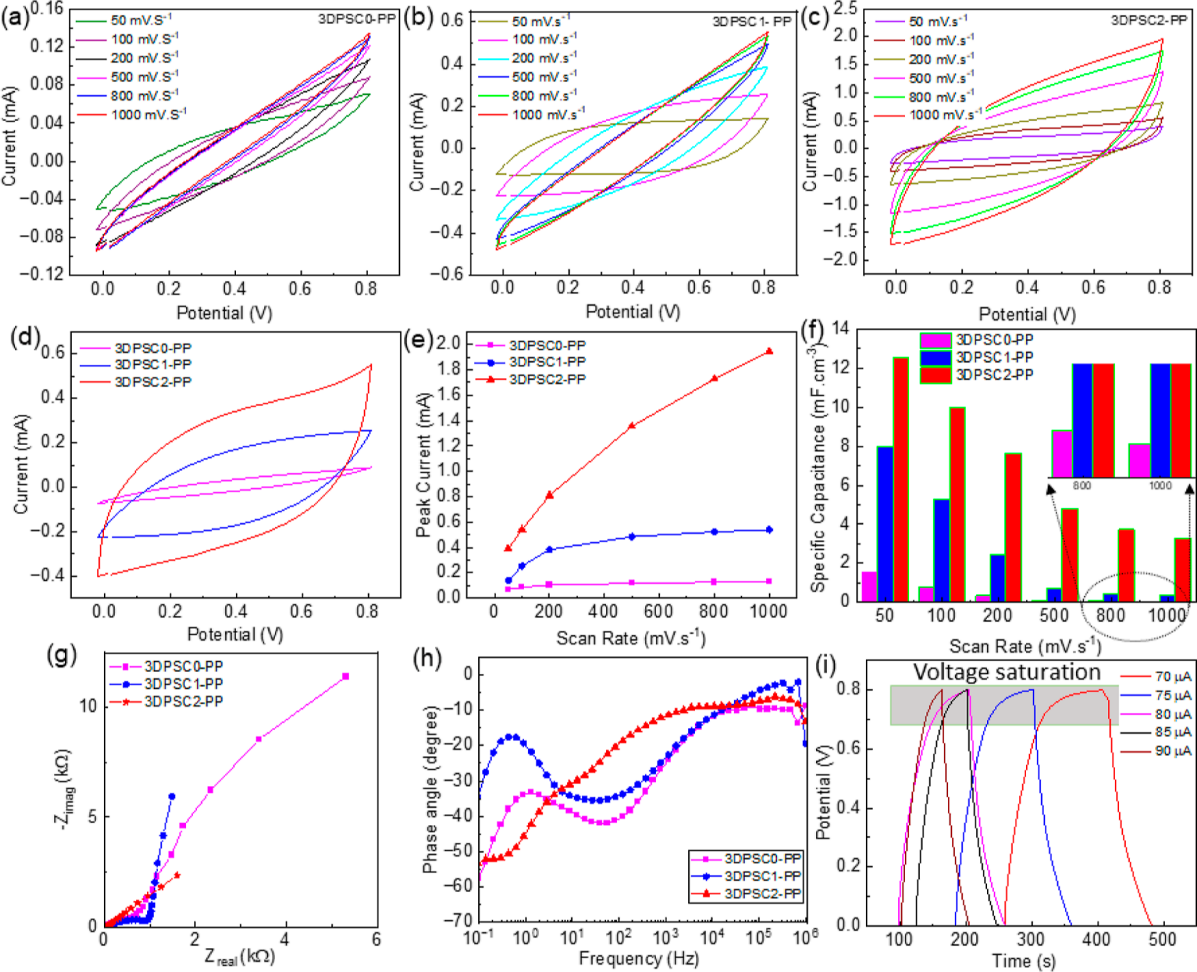
(a–c) CV curves with different scan rates for 3DPSC0-PP, 3DPSC1-PP, and 3DPSC2-PP, respectively. (d) Comparison of CV curves observed for 3DPSCs-PP at 100 mV·s^–1^. (e) Peak current with scan rate for various 3DPSCs-PP. (f) Specific capacitance was measured from CV curves with different scan rates for various 3DPSCs-PP. (g,h) Nyquist and Bode plot for 3DPSC0-PP, 3DPSC1-PP, and 3DPSC2-PP. (c–e) GCD plots 3DPSC2-PP for different currents.

Figure 6a–c shows the CV data at a different scan rate for the fabricated 3DPSCs-PP. Similar to 3DPSCs, the analysis of 3DPSC-PP exhibited a quasi-rectangle shape due to the pseudocapacitance reaction. Compared with 3DPSC0-PP and 3DPSC1-PP, the 3DPSC2-PP exhibits a larger area in the CV curve at 100 mV·s^–1^ as illustrated in Figure 6d. For the devices, we measured the peak current at 0.8 V, and it increased with the increasing scan rate, as illustrated in Figure 6e. It was found that the polymer coating remarkably enhances the current rate and hence energy storing performances for the porous electrode. The specific capacitance of 3DPSCs-PP measured from the CV analysis for various scan rates is given in Figure 6f. Figure 6f reveals that the 3DPSC2-PP has a specific capacitance of 12.55 mF·cm^–3^ at 50 mV·s^–1^ and is 8 times higher than that for samples without porous electrodes. The EIS analysis for the 3DPSCs-PP shown in Figure 6g reveals that the solid electrode with conducting polymer coating has very high ionic resistance in a lower frequency range compared to porous electrodes coated with polymers. The ESR values of the fabricated 3DPSC0-PP, 3DPSC1-PP, and 3DPSC2-PP are 24.84, 39.54, and 13.41 Ω, respectively, and 3DPSC2-PP illustrates lower contact resistance. The 3DPSC1-PP shows higher resistance and has a lower phase angle at low frequency in the Bode plot (Figure 6h). The Bode plot illustrates different behavior in the curve for 3DPSC2-PP as compared with 3DPSC0-PP and 3DPSC1-PP due to the electrode reaction.

Finally, GCD analysis was carried out for the devices, and it was observed that 3DPSC2-PP required a high current for charging and discharging compared to the other two devices. GCD curves for 3DPSC2-PP (for the second cycle) for different applied currents are shown in Figure 6i. It was found that the charging–discharging using a lower current device illustrates a saturation voltage. To reach the voltage of 0.8 V without any saturation, the device requires a high current. However, the 3DPSC0-PP and 3DPSC1-PP required lower current (40 μA) for GCD analysis which is given in Figure S5a,b in Supporting Information. The specific capacitance of the 3DPSC2-PP measured from the GCD curve is 24.42 mF·cm^–3^ at an applied current of 70 μA. The device has energy and power densities of 1.98 μW h·cm^–3^ and 107.85 μW·cm^–3^, respectively. It was observed that the 3DPSC2-PP can operate at a higher current compared with 3DPSC0-PP and 3DPSC1-PP. This leads to comparatively higher performance of 3DPSC2-PP. However, the Coulombic efficiency of the device is lower for low current GCD analysis (44.5% at 70 μA). The variation of the Coulombic efficiency of 3DPSC2-PP is given in Figure S6 in Supporting Information.

### 3.4 Comparison of Performances of 3DPSC and 3DPSC-PP

**Figure 7:**
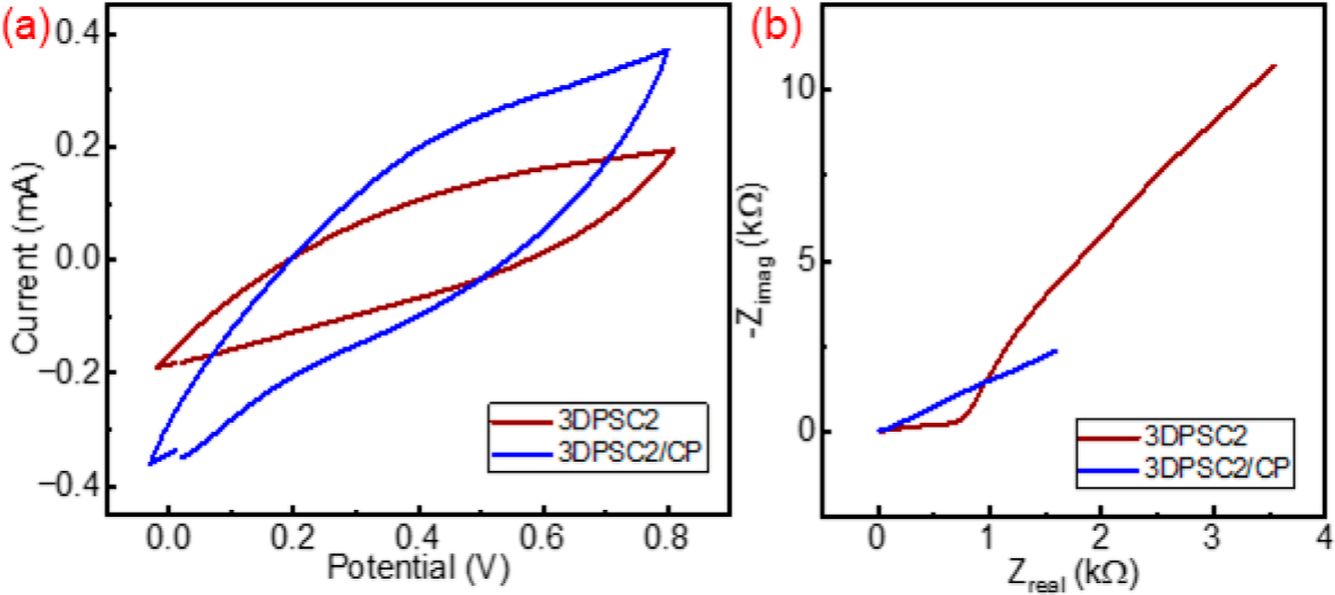
Comparison of (a) CV curve at 100 mV·s^–1^ and (b) Nyquist plot for 3DPSC2 and 3DPSC2-PP.

The performances of 3DPSCs illustrate that compared with the solid electrode, the porous electrode (3D2) illustrates high capacitance and is almost four times higher than the solid electrode (3D0) at 50 mV·s^–1^. Similarly, in 3DPSC-PP, the capacitance of 3DPSC2-PP is 8 times higher than that for 3DPSC0-PP. The surface modification and mass loading with PEDOT:PSS presented a significant performance change. A comparative study was carried out for 3DPSC2 and 3DPSC2-PP and Figure 7a illustrates the comparison of the CV curve at 100 mV·s^–1^. The CV and EIS (Figure 7b) analyses show that the conducting polymer coating on the top of the porous electrode significantly enhances the electrochemical performance. A strong reduction in resistance from EIS reveals the more ionic diffusion on the electrode. The CV analysis illustrates that at 100 mV·s^–1^, the specific capacitance of 3DPSC2-PP has a capacitance of 10.005 mF·cm^–3^ and is four times higher than that of 3DPSC2. Moreover, this reduction in surface resistance of the electrode reduces the voltage drop in 3DPSC2-PP. For 50 μA applied current the 3DPSC2 exhibits a voltage drop of 0.21 V, however, the 3DPSC2-PP in a higher current of 70 μA illustrates a low voltage drop of 0.036 V. This reduction in voltage drop also causes an almost twofold energy density enhancement as compared with uncoated electrodes.

The energy storage capabilities of various electrode materials are presented in Table 1. It is clear that the choice of materials used for the fabrication of 3D-printed electrodes significantly influences the energy-storing performance. Compared with these reports, in this work, we investigated how the cellular porous structure of a 3D printed electrode and its interaction with the conductive polymer influence electrochemical performances. Comparing the results to similar studies, variations in electrochemical performance, such as capacitance, energy density, and power density, can be attributed to differences in active electrode materials and electrolytes. The mass loading of pseudocapacitive or conductive fillers significantly impacts energy storage performance, and further investigations will be conducted in our future research. The performance of these devices underscores their potential applications in portable electronics and structural energy storage devices. Leveraging 3D printing for structural electrode fabrication will facilitate the development of distributed energy storage devices.

**Table 1:** Comparison of Performances of Various 3D-Printed Electrode-Based SCs

material	electrolyte	voltage window (V)	specific capacitance	energy density	power density	lifecycle	ref
CoNi_2_S_4_/NiCo-LDHs	KOH	0–0.5	28.71 F cm^–3^ at 10 mA cm^–3^	0.582 mW·h·cm^–3^	85.81 mW·cm^–3^	75.2% (1000)	(35)
BPNS/PPy	EMI-TFSI	0–0.6	417 F·g^–1^ at 0.2 A·g^–1^	6.5 W·h·kg^–1^	0.0374 W·kg^–1^	87% (10 000	(36)
N-carbon@graphene/MnO_2_//N-carbon@graphene/MoS_2_	PVA/LiCl	0–1.8	318.82 mF·cm^–2^ at 0.5 mA·cm^–2^	143.15 μW h·cm^–2^	450.02 μW·cm^–2^	98.63% (12 000)	(37)
GO/Fe_3_O_4_/CNT/PVA	PVA/H_2_SO_4_	0–1	2.9 F·cm^–2^ at 0.4 A·g^–1^	0.13 mW·h·cm^–2^	285 mW·cm^–2^	98.5% (10 000)	(38)
AC/graphite/CNF	CNF/glycerol/NaCl	–0.5–0.5	25.6 F·g^–1^ at 1 mV·s^–1^	0.88 W·h·kg^–1^	830 W·kg^–1^	99% (2000)	(39)
MoS3–x@nanocarbon//Ti_3_C2Tx	PVA/H_2_SO_4_	0–1	55.60 mF·cm^–2^ at 0.26 mA cm^–2^	56.94 μW·h·cm^–2^	6.00 mW·cm^–2^	92.8% (25 000)	(40)
NiCo2O_4_/graphite//N-doped carbon/graphite	PVA/KOH	0–0.6	0.81 F cm^–3^ at 75 mA·cm^–3^	36.9 W·h·kg^–1^	20 kW·kg^–1^	87.8% (10 000)	(41)
WCF-N@ZnCoSe2-MXene	thermosetting polymer electrolyte	0–1	19.36F·g^–1^	2.69 W·h·kg^–1^	43.20 W·kg^–1^	83.7% (6000)	(42)
NiCo-MOF@CoOOH@V2O5	KOH-poly(vinyl alcohol) (PVA) gel	0–1.5	585 mF·cm^–2^	159.23 μW·h·cm^–2^	0.34 mW·cm^–2^		(43)
PL HG Precursor Solution	PAAm/H_2_SO_4_ gel	0–1	74.76 mF·cm^–3^			74.1% (3000)	(44)
PEEK/MWCNT- PEDOT: PSS (pore structure-3DPSC2)	KOH	0–0.8 (0.764 after drop)	24.42 mF·cm^–3^	1.98 μW·h·cm^–3^	107.85 μW·cm^–3^		This work

## 4 Conclusions

In this study, we realized a 3D-printed supercapacitor (3DPSC) employing a composite material, composed of MWCNTs and PEEK. Utilizing the FFF method, we processed PEEK/MWCNT composites to create three distinct 3D-printed electrodes: one with a surface area density of 1.2 mm^–1^ and two periodically porous electrodes with surface area densities of 7.3 and 7.1 mm^–1^, respectively. Employing these electrodes, we constructed three unique 3DPSCs, optimizing their energy storage capabilities through the application of a PEDOT:PSS coating. Electrochemical investigations revealed that the carefully controlled porosity of the 3D-printed electrodes led to significantly improved capacitive properties. The most significant performance change was observed for the device utilizing a cellular electrode with a surface area density of 7.1 mm^–1^, coated with PEDOT:PSS. This device exhibited a specific capacitance of 24.42 mF·cm^–3^ at an applied current of 70 μA, accompanied by energy and power densities of 1.98 μW h·cm^–3^ and 107.85 μW·cm^–3^, respectively. The application of the conductive polymer coating on the 3D-printed electrode not only enhanced capacitance but also mitigated the voltage drop. This study underscores the benefits of using porous electrodes in energy storage devices, such as enhanced mass loading of high-performance materials, efficient ion diffusion and electron transport, and accelerated reaction kinetics. However, the device showed a lower energy density compared to other metal oxide-based 3D-printed electrodes. Additionally, this electrode presents limitations for use in flexible electronics applications. Future designs incorporating new high-energy-storing materials, such as metal oxide-polymer composites in architected porous electrodes, hold promise for further enhancing the performance of additive manufacturing-enabled energy storage devices.

## Supporting Information Available

Material morphological and electrochemical characterization; SEM images of PEDOT: PSS coated porous electrodes and CV and GCD data and analysis of different SCs fabricated; (PDF)

## Abbreviations

SC: supercapacitor
PEDOT: PSS-poly(3,4-ethylenedioxythiophene) polystyrene
PEEK: polyether ether ketone
MWCNTs: multiwalled carbon nanotubes
FFF: fused filament fabrication
