# Supplementary material for: Enhancing Supercapacitor Electrochemical Performance with 3D Printed Cellular PEEK/MWCNT Electrodes Coated with PEDOT: PSS
Source: ACS Omega. 2024 Jul 23;9(31):33998–4007. doi: 10.1021/acsomega.4c04576 (PMC11307982; doi:10.1021/acsomega.4c04576)
Supplement: Supplementary file 1 — ao4c04576_si_001.pdf [file ao4c04576_si_001.pdf]

Supporting Information

# Enhancing Supercapacitor Electrochemical Performance with 3D Printed Cellular PEEK/MWCNT Electrodes Coated with PEDOT: PSS

*Athul C. S. Chandran[a], Johannes Schneider[b], Reshma Nair[a], Buchanan Bill[a], Nikolaj Gadegaard[b], Richard Hogg[b,c], Shanmugam Kumar[b]*, Libu Manjakkal[a]**

[a]School of Computing and Engineering & the Built Environment, Edinburgh Napier University, Merchiston Campus, EH10 5DT, UK.

[b]James Watt School of Engineering, University of Glasgow, Glasgow G12 8QQ, UK.

[c]School of Engineering and Applied Science, Aston University, B4 7ET Birmingham, UK

Correspondence Email: msv.kumar@glasgow.ac.uk; L.Manjakkal@napier.ac.uk

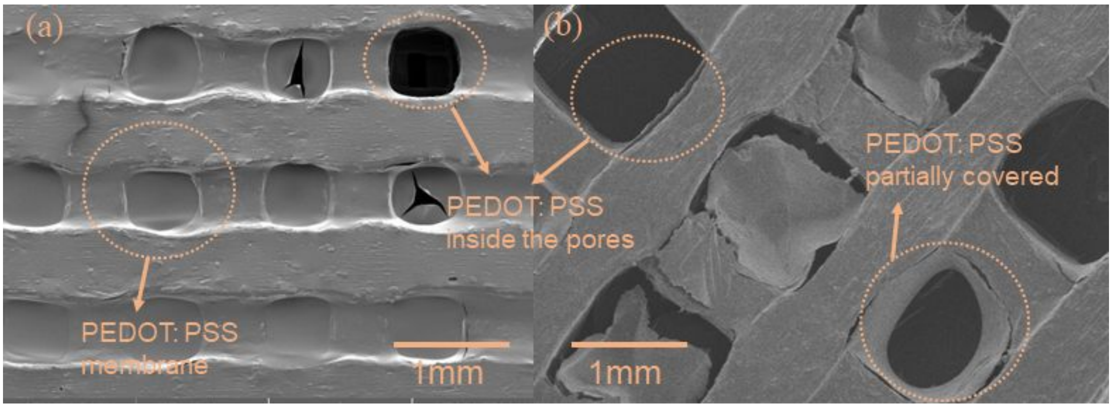


Fig. S1: (a) and (b) SEM images of PEDOT: PSS coated 3D printed porous electrode.

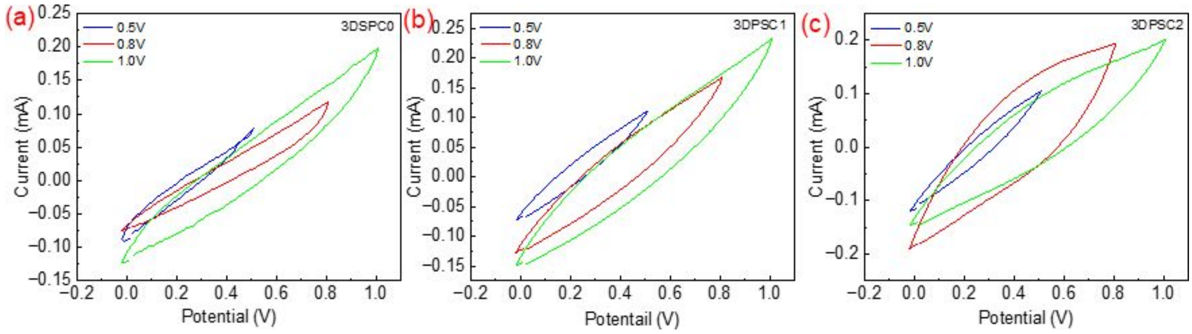


Fig. S2: (a) – (c) CV curves for 3DPSC0, 3DPSC1, and 3DPSC2 for different potential windows.

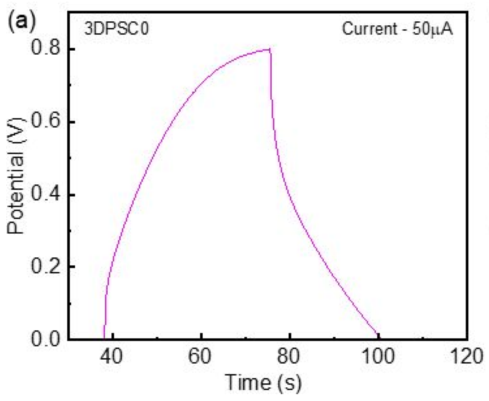


Fig. S3: GCD plot for (a) 3DPSC0 and (b) 3DPSC1 at 50 µA.

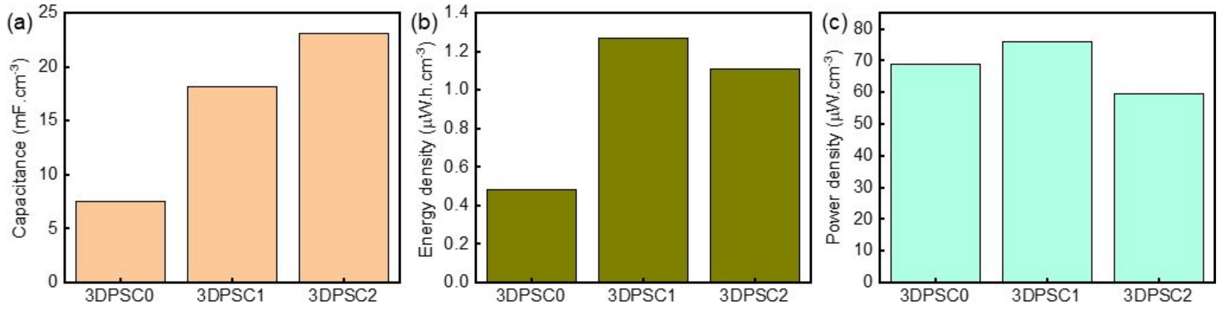


Fig. S4: (a) –(c) Specific capacitance, Energy and Power density variations for 3DPSCs

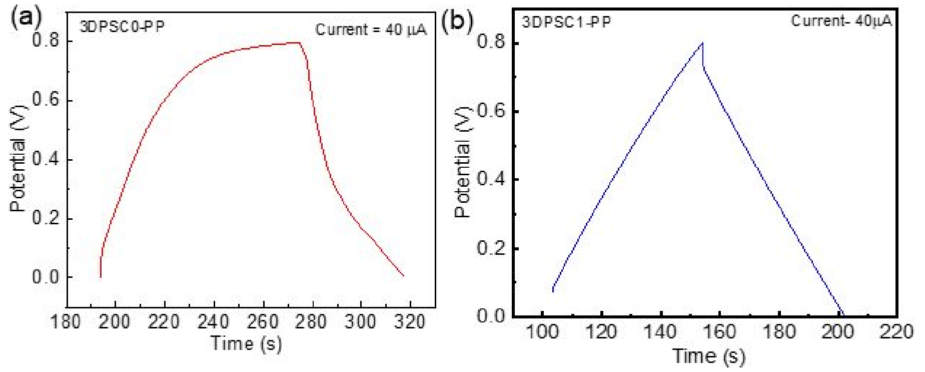


Fig. S5: (a) and (b) GCD curves for 3DPSC0-PP and 3DPSC1-PP at 40 µA applied current.

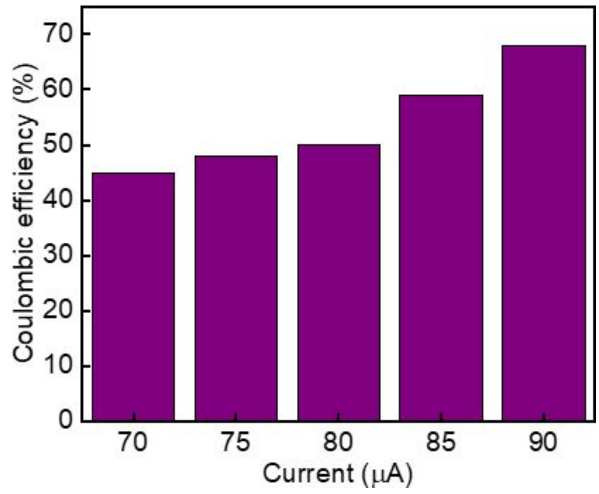


Fig. S6: The changes in Coulombic efficiency of 3DPSC2-PP for various applied currents.
